# Neurorehabilitation From a Distance: Can Intelligent Technology Support Decentralized Access to Quality Therapy?

**DOI:** 10.3389/frobt.2021.612415

**Published:** 2021-05-05

**Authors:** Olivier Lambercy, Rea Lehner, Karen Chua, Seng Kwee Wee, Deshan Kumar Rajeswaran, Christopher Wee Keong Kuah, Wei Tech Ang, Phyllis Liang, Domenico Campolo, Asif Hussain, Gabriel Aguirre-Ollinger, Cuntai Guan, Christoph M. Kanzler, Nicole Wenderoth, Roger Gassert

**Affiliations:** ^1^ Rehabilitation Engineering Laboratory, Department of Health Sciences and Technology, ETH Zurich, Switzerland; ^2^ Future Health Technologies, Singapore-ETH Centre, Campus for Research Excellence and Technological Enterprise (CREATE), Singapore, Singapore; ^3^ Neural Control of Movement Laboratory, Department of Health Sciences and Technology, ETH Zurich, Switzerland; ^4^ Centre for Advanced Rehabilitation Therapeutics, Tan Tock Seng Hospital Rehabilitation Centre, Singapore, Singapore; ^5^ Rehabilitation Research Institute Singapore, Nanyang Technological University, Singapore, Singapore; ^6^ Singapore Institute of Technology (SIT), Singapore, Singapore; ^7^ School of Mechanical and Aerospace Engineering, Nanyang Technological University, Singapore, Singapore; ^8^ Articares Pte Ltd, Singapore, Singapore; ^9^ School of Computer Science and Engineering, Nanyang Technological University, Singapore, Singapore

**Keywords:** neurorehabilitation, robot-assisted therapy (RAT), clinical intelligence, decentralized care, stroke

## Abstract

Current neurorehabilitation models primarily rely on extended hospital stays and regular therapy sessions requiring close physical interactions between rehabilitation professionals and patients. The current COVID-19 pandemic has challenged this model, as strict physical distancing rules and a shift in the allocation of hospital resources resulted in many neurological patients not receiving essential therapy. Accordingly, a recent survey revealed that the majority of European healthcare professionals involved in stroke care are concerned that this lack of care will have a noticeable negative impact on functional outcomes. COVID-19 highlights an urgent need to rethink conventional neurorehabilitation and develop alternative approaches to provide high-quality therapy while minimizing hospital stays and visits. Technology-based solutions, such as, robotics bear high potential to enable such a paradigm shift. While robot-assisted therapy is already established in clinics, the future challenge is to enable physically assisted therapy and assessments in a minimally supervized and decentralized manner, ideally at the patient’s home. Key enablers are new rehabilitation devices that are portable, scalable and equipped with clinical intelligence, remote monitoring and coaching capabilities. In this perspective article, we discuss clinical and technological requirements for the development and deployment of minimally supervized, robot-assisted neurorehabilitation technologies in patient’s homes. We elaborate on key principles to ensure feasibility and acceptance, and on how artificial intelligence can be leveraged for embedding clinical knowledge for safe use and personalized therapy adaptation. Such new models are likely to impact neurorehabilitation beyond COVID-19, by providing broad access to sustained, high-quality and high-dose therapy maximizing long-term functional outcomes.

## Introduction

Stroke is a leading cause of disability and morbidity globally, accounting for 132 million disability-adjusted life-years (DALYs) worldwide (GBD, 2018). Among others, the neurological damage resulting from a stroke can lead to severe upper limb sensorimotor impairment, affecting a person’s ability to work and take part in activities of daily living. To date, there is no cure for stroke and patients rely on neurorehabilitation services long after their injury to at least partially recover sensorimotor function.

Currently, neurorehabilitation strongly relies on physical and occupational therapy sessions, which are primarily based on one-to-one interactions with healthcare practitioners either during an inpatient hospital stay (mostly during the acute to sub-acute phase) or as part of regular visits to specialized outpatient institutions (mostly during the sub-acute to chronic phase). This current model of care is highly resource demanding and already faces important challenges to cope with constantly increasing numbers of patients due to changing demographics, shortage of trained healthcare providers, and economic pressure to minimize healthcare costs. As a result, therapy dose is typically rather low at all stages of the continuum of care, despite the growing evidence that intensive high-dose neurorehabilitation positively impacts sensorimotor function even long after the injury (Daly et al., 2019; Ward et al., 2019).

The coronavirus (COVID-19) pandemic brought additional critical constraints to this already fragile ecosystem (Caso and Federico, 2020). Patients after stroke belong to the population at risk (Jordan et al., 2020) and essential care, such as neurorehabilitation services, was set to lower priority to avoid overloading the healthcare system. Furthermore, physical distancing measures and the cut-back on face-to-face consultations have led to a reduction in clinic and therapy stays/visits. As a result, several studies have reported that the quality of care in stroke patients has been impacted (Bersano et al., 2020; De Sousa et al., 2020; Richter et al., 2021). A recent survey revealed that a large majority of healthcare professsionals involved in stroke care (primarily neurologists, interventionalists and rehabilitation physicians) were concerned that this lack of care will have had a noticeable negative impact on long-term outcomes, and that rehabilitation is likely the most affected area of stroke care (De Sousa et al., 2020).

COVID-19 crystalizes the limitations of existing healthcare models and highlights the urgent need to rethink conventional neurorehabilitation so that high-quality therapy can be provided while the need for hospital stays and visits is minimized. In the last decade, the digital revolution has fueled the vision of new scalable technologies that provide higher doses of high-quality rehabilitation along the continuum of care and, in particular, in the home of patients (Galea, 2019). However, such solutions for “neurorehabilitation from a distance” still remain in their infancy (Dafer et al., 2020; De Sousa et al., 2020) and have so far only been investigated as separate elements (i.e., individual technologies and therapy concepts). This article aims to provide an overarching vision on how different existing technological solutions could be combined in the form of a connected RehabGym. The overall goal of such a framework is to promote recovery, maintain functional gains and maximize independence by optimally using the potential of rehabilitation technology. For this, we propose concrete considerations for the implementation of minimally supervized robot-assisted therapy as a possible approach to provide quality, high-dose neurorehabilitation solutions along the continuum of care. We argue that user-friendly, intelligent and robust technology could help transform the current hospital-centered model into a home-centered model of care that is potentially more resource- and cost-effective, and robust to extreme situations such as the COVID-19 pandemic.

## Neurorehabilitation From a Distance

### From a Hospital-Centered Model to a Home-Centered Model of Neurorehabilitation

**FIGURE 1:**
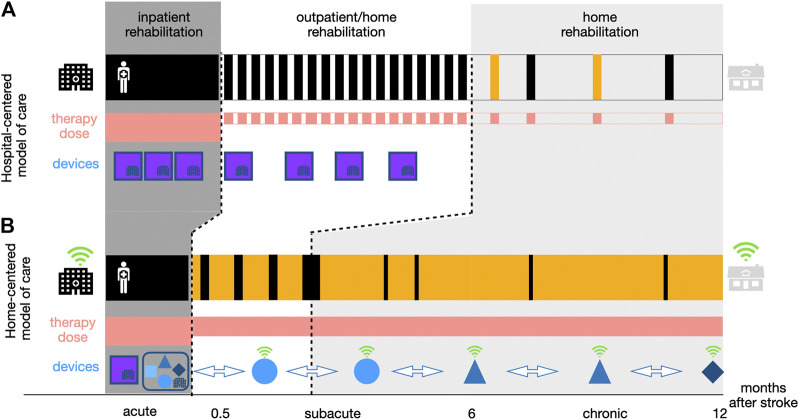
Two approaches to neurorehabilitation along the continuum of care. Compared to the hospital-centered model of stroke rehabilitation (a), the home-centered model of care (b) aims to reduce the time a patient spends in a healthcare institution (depicted in black) physically visiting a rehabilitation professional. However, patients receive a similar or even potentially higher dose of therapy (depicted in red) due to continued self-directed training at home (depicted in yellow). This should be supported by different complementary mobile technology-based devices (e.g., robotics, wearables, virtual reality games) (blue shapes) introduced early in the inpatient rehabilitation (i.e., RehabGym), and that can, after a familiarization phase under therapist supervision, be taken home by patients to continue with a minimally supervized rehabilitation training (i.e., without the presence of a clinician or expert operator). These devices should be intelligent connected tools (depicted in green, more detail on a possible implementation in *Going Beyond COVID-19: Moving Towards Minimally-Supervised Robot-Assisted Therapy*) allowing for remote patient monitoring, while empowering and motivating patients to engage in high-quality therapy from a distance, which is not possible with traditional stationary neurorehabilitation technologies (purple blocks).

Neurorehabilitation after stroke often starts at the bedside early after the incident (e.g., after 2–3 days once the patient is stable), is then continued for 1–3 months in a rehabilitation clinic and later transitions to community-based rehabilitation treatment, for example, in an outpatient center (i.e., 5–6 weeks of intensive training). While such a model allows healthcare practitioners to closely monitor their patients and support them and their families (both physically and emotionally) throughout the recovery process, it heavily relies on physical access to medical facilities and sustained interactions with trained specialists (hospital-centered model, Figure 1A). This neurorehabilitation model is not only challenged by events such as the COVID-19 pandemic but also by changing demographics, which might lead to a shortage of human resources and non-sustainable costs. This ultimately affects the dose and quality of therapy patients receive, limiting rehabilitation to intense but relatively short and early time periods that might be insufficient to achieve functional recovery. After discharge, there is typically limited support for continued care management or to motivate patients to self-engage in physical activities or rehabilitation exercises at home, which are necessary for the maintenance of functional gains (Nicholson et al., 2013). This gap may explain the often observed decrease in functional ability or learned nonuse of the impaired limb (Taub et al., 2006; Hidaka et al., 2012).

To offer a more sustainable approach, there is a need to shift the existing hospital-centered model of care toward a more home-centered model (Figure 1B). In such a scheme, selected patients are discharged from hospital/outpatient centers earlier and provided with various solutions to perform high-quality therapy at home. This facilitates decreased dependence on hospital fixed schedules and limited resources, thereby bringing the promise of increasing the overall therapy dose patients may receive, provided that they engage in self-directed therapy.

While a home-centered approach to conventional therapy is not new and has been shown to be implementable in a cost-efficient way (Mayo et al., 2000; Hillier and Inglis-Jassiem, 2010; Mayo, 2016), digital technologies and artificial intelligence have a key role to play in further establishing and supporting this model, as they could provide patients with intelligent connected tools empowering and motivating them to engage in quality therapy from a distance.

### Technologies Supporting Neurorehabilitation From a Distance

A variety of technological approaches have been proposed to support the implementation of neurorehabilitation from a distance, and new developments emerged during the COVID-19 pandemic as possible answers to the limited access to medical facilities. Existing solutions span from simple webinars or mobile phone applications informing patients, to chatbots (i.e., artificial intelligent coaches (Argent et al., 2018; Tudor Car et al., 2020)) demonstrating and encouraging home-based exercises, or virtual reality (VR) exergames sometimes supported by passive instrumented tools (e.g., orthoses, gloves or objects to manipulate) (Nijenhuis et al., 2017). Telerehabilitation (or telemedicine) has already been widely studied as a method to support neurorehabilitation from a distance (Tyagi et al., 2018; Kuah et al., 2019; Laver et al., 2020) and has often been presented as a possible answer to meet neurorehabilitation needs during the COVID-19 pandemic (Chang and Boudier-Revéret, 2020; Turolla et al., 2020). In a typical telerehabilitation scenario, healthcare practitioners interact with patients over a live communication stream, offering the possibility to guide and encourage patients while monitoring their progress. Telerehabilitation approaches that go beyond video/audio support and offer additional connected hardware (e.g., USB-based wrist blood pressure cuff and mat with contact sensitive switches, gaming driving wheel with a special gripper, joysticks, etc.) have been proposed for stroke patients (Johnson et al., 2007; Dodakian et al., 2017; Johnson et al., 2017). Telerehabilitation using socially assistive robots (e.g., a humanoid robot with telepresence and computer vision under the supervision of a remote clinician) can also deliver emotional support and help to increase the patient’s motivation (Fasola and Mataric, 2010; Pulido et al., 2019; Sobrepera et al., 2020). Nevertheless, the lack of physical assistance, an essential facilitator for movement therapy in patients with sensorimotor impairments, and the inability to actively measure physiological parameters for providing feedback to improve performance strongly limit such telehealth approaches. Also, most telerehabilitation applications still rely on the synchronous presence and supervision of a rehabilitation professional, thereby not solving the underlying issue of lacking resources for neurorehabilitation.

Active technologies such as robotics, which can guide and assist motion while collecting objective measures of movement quality, might be the key enabler providing access to quality therapy from a distance, without the need for constant supervision by a therapist or expert operator on site. Technology-assisted therapy has been established as a tool to complement conventional rehabilitation in the clinics, with the ability to safely deliver high therapy dose and intensity in suitably selected patients (Veerbeek et al., 2017; Gassert and Dietz, 2018; Mehrholz et al., 2020). For the upper limb, there is increasing evidence demonstrating that robot-assisted therapy is at least as good as usual care (Ranzani et al., 2020) and provides the unique ability to actively assist patients with physical impairments and objectively monitor performance and engagement through objective sensor measurements. Nevertheless, most rehabilitation robots today remain complex systems (e.g., multi-degrees-of-freedom exoskeletons, or devices requiring precise attachment/positioning of the user, or systems relying on complex graphical user interface where input from an operator is required) that have so far been limited to supervised use in the clinic with close one-to-one monitoring and readily available technical assistance.

## Going Beyond COVID-19: Moving Toward Minimally Supervized Robot-Assisted Therapy

To fully exploit the potential of rehabilitation technologies such as robotics and truly revolutionize neurorehabilitation delivery, there is a need to move out of the research labs or clinical settings. While few pilot studies have demonstrated feasibility of home self-administered upper extremity training with robots (Sivan et al., 2014; Wolf et al., 2015), this translation remains a major challenge due to multiple key technical and clinical requirements imposed by minimally supervized use.

### Technical Requirements and Usability

Realistically, for a rehabilitation robot to be adopted and regularly used by neurological patients in their home environment, it should be: intrinsically safe and user-friendly, portable for easy deployment in homes where available space might be scarce, robust so that little to no maintenance is needed over potentially long periods of use, and scalable (low-cost and relying on typically already available resources in patients’ home such as standard electrical and internet connections). Of these technical aspects, ease of use in an independent way is probably the most critical point to ensure acceptance and adoption, and a point that has been so far rarely evaluated in existing robotic technologies for neurorehabilitation (Zhang et al., 2011; Catalan et al., 2018).

Usability considerations (e.g., in the form of user-centered design involving patients in the development process (Meyer et al., 2019)) should be taken into account not only at the level of the hardware (e.g., how to turn it on/off, how to don/doff a device, etc.) but also at the level of the software and graphical user interface, which should be intuitive and easy to navigate for non-experts in computer use, or patients with cognitive deficits (Ranzani et al., 2021). User archetypes created through data generated from actual target users might increase the potential for better design of technology-assisted interventions in stroke rehabilitation (Haldane et al., 2019). Along the same lines, robot-assisted exercises should be easily understandable to ensure safe use and the training of physiologically meaningful exercises.

### Clinical Artificial Intelligence

Besides technical requirements, it is fundamental to integrate clinical knowledge into technologies to be used at home in a minimally supervized way. We define here clinical artificial intelligence (cAI) as a combination of medical, psychological and technical knowledge in the form of embedded algorithms analyzing and processing online the data generated by digital technologies. As such, cAI is expected to play a key role in clinical decision making, online adaptation of therapy exercises, and monitoring of progress through the extraction of validated assessment scores (Kanzler et al., 2020). For example, in a typical minimally supervized robot-assisted rehabilitation scenario, it is envisioned that an initial therapy plan (e.g., combination of exercises at a specific dose and intensity in order to achieve selected goals) is established by a therapist during the initial inpatient rehabilitation. However, this initial therapy plan should be able to adapt and evolve based on patients’ progress without requiring the direct intervention of the therapist. Algorithms have been proposed, where cAI-based decisions regarding exercise selection were suggested to therapists during supervised rehabilitation in chronic stroke patients, showing strong agreement with therapist perception and decisions (Panarese et al., 2012). In a similar way, several assessment-driven therapy adaptation algorithms of various complexity (e.g., based on online extraction of performance biomarkers, or including machine learning models, etc.) have been proposed and evaluated with stroke patients to tailor rehabilitation exercises to the ability and needs of each patient. Several promising studies, under supervised use in the clinic, validated the feasibility of such algorithms to dynamically adapt difficulty and intensity of therapy exercises on a trial-per-trial basis, thereby ensuring that the therapy remains at an optimal level of challenge to maintain motivation for long term adoption (Metzger et al., 2014; Giang et al., 2020). Finally, cAI algorithms should as well monitor progress on a daily basis and detect potential decline in use and/or performance, with the possibility to feed this information back to the patients and caregivers. In a similar way, cAI should detect undesired symptoms that could lead to pain, increase in muscle tone, or upper limb compensatory movements, which could also be objectively extracted from the collected sensor data (Wittmann et al., 2016; Ranzani et al., 2019; Cai et al., 2020). When a severe abnormal deviation from the normal movement range/trajectories is detected, the cAI should initiate a safety procedure to prevent suboptimal rehabilitation outcomes or, in the worst case, an injury. It should be emphasized that the objective of cAI is not to replace healthcare practitioners, but to support a home-centered model of care where one-to-one presence of therapists is not viable. Communication channels should nevertheless be in place to asynchronously inform clinicians about therapy status and potential deviations e.g., via digital alerts, through the generation of daily reports, or via short digital questionnaires filled by patients/caregivers (de Jong et al., 2014; Hill & Breslin, 2016; Kowatsch et al., 2019). Once an unexpected event during minimally supervized training occurred, the cAI should alert the therapists in the hospital/clinic so that (tele)consultation can be arranged to evaluate the cause and to ensure that appropriate advice can be provided to the patient. These measures will aid in mitigating the risks of injuries.

### Towards a Connected RehabGym

**FIGURE 2:**
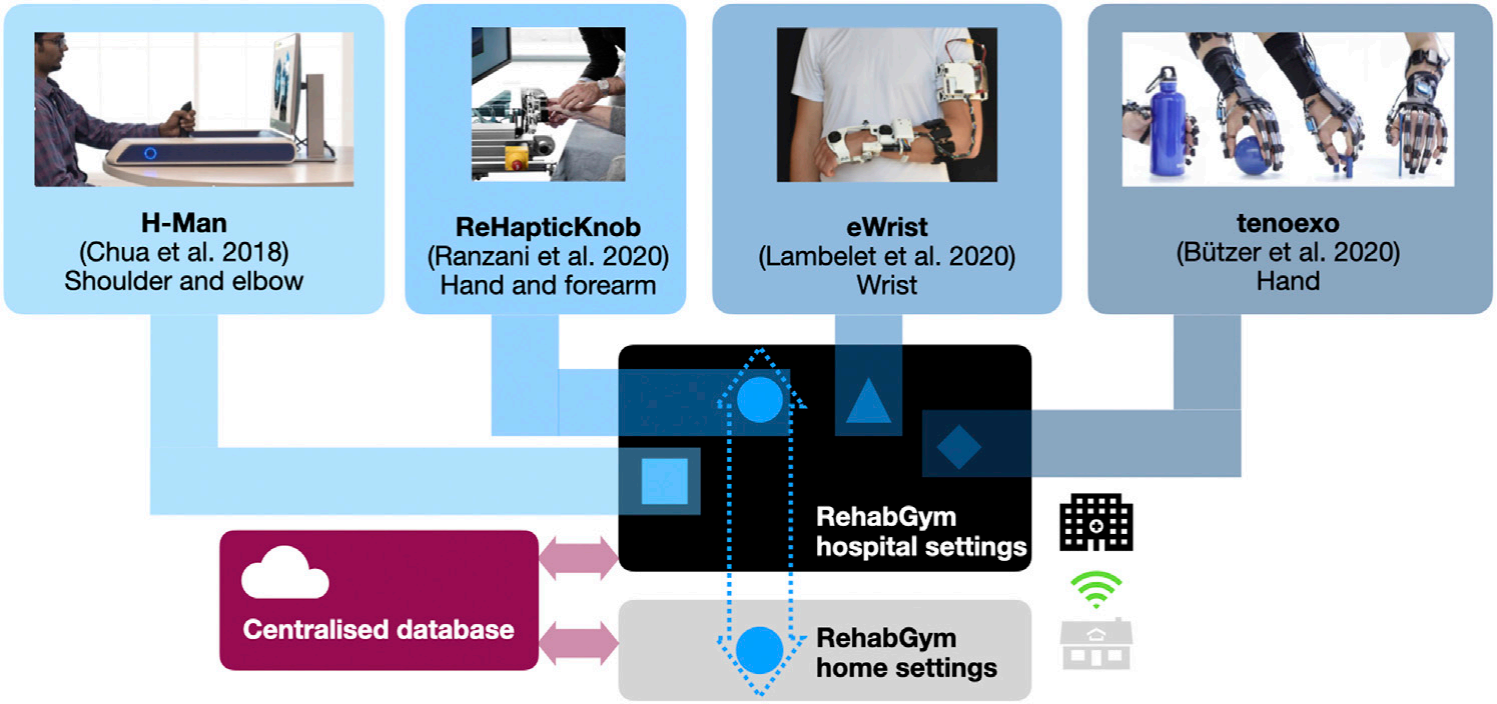
Conceptual overview of a connected RehabGym, with examples of user-friendly and complementary (i.e., targeting all segments of the upper limb) mobile robotic technologies for minimally supervized neurorehabilitation. All technologies are first introduced during inpatient rehabilitation at the hospital, and selected technologies (e.g., the one(s) best adapted to the impairment level and rehabilitation goals of a patient) are taken home upon discharge. Connected devices ensure asynchronous (i.e., not online/real-time), remote communication with healthcare professionals for monitoring purposes.

A robotic device for minimally supervized rehabilitation combined with cAI bears high potential for treating selected patients. However, as for any therapy, a more holistic approach is needed since patients with various impairment levels will have different needs and therapy goals that could hardly be met by a single device. The transition to a technology-supported home-centered model of care may therefore rely on a network of modular rehabilitation technologies that are complementary and interconnected within a common digital therapy platform. In this ideal concept of a connected “RehabGym”, we foresee simple, dedicated, rehabilitation technologies targeting different body segments (e.g., shoulder/elbow, wrist, hand), motor function (e.g. reaching, grasping, haptic exploration) and impairment level (from hemiparetic to highly functional patients) being proposed as a battery of digital interventions available for the treatment of a patient. A common digital therapy platform offers the unique advantage of sharing user-interaction features between devices, to improve usability and help seamlessly transition from one device to the other (e.g., within a therapy session, or over the course of rehabilitation). Figure 2 presents a possible set of selected user-friendly, mobile, complementary robotic systems targeting different components of the upper limb, that could be used as a basis to implement such a connected RehabGym concept (Chua et al., 2018; Butzer et al., 2020; Lambelet et al., 2020; Ranzani et al., 2020; Ranzani et al., 2021).

One possible implementation avenue would be to first deploy the RehabGym technologies in hospital settings, where a patient could familiarize with the use of each technology during the essential inpatient rehabilitation (under supervision), and where rehabilitation practitioners could identify which technologies are most likely to benefit a patient. This initial supervised step is certainly fundamental to ensure safe use of a robotic technology and adoption by the user. In the clinic, such a room equipped with multiple complementary devices could be operated with a single therapist supervising multiple patients, which should provide a cost-effective solution (Hesse et al., 2014; Bustamante Valles et al., 2016) and a compatible approach to minimized one-to-one interactions.

Upon discharge, a patient could then take home a selected rehabilitation technology to continue therapy in a minimally supervized manner. If available, the patient’s caregiver(s)/family will also be instructed on how to operate the device(s) safely and how to optimally support the rehabilitation process (e.g., support setting up devices and promoting motivation and compliance). Caregivers and family could play an essential role in assisting patients who are not so familiar with handling devices or suffer from more severe impairments, thereby ensuring a successful transfer of the RehabGym to the home environment. Cloud computing should enable data exchange to a centralized database where patient profiles (e.g., data from all RehabGym technologies used by the patient, collected digital health biomarkers, as well as clinical assessment data) are stored and can be accessed remotely by a cAI concept shared by all devices of the connected RehabGym. It should be noted that the system should be operational even if the internet connection is slow or instable, which can be ensured by asynchronous data exchange and storage on the cloud. The cloud for the RehabGym should be securely hosted either within the hospital IT infrastructure or by a third party who is accountable for information security (e.g., data confidentially, integrity and availability). Algorithms should update therapy plans and other training parameters (e.g., suggest transfer to another RehabGym device/exercise), and inform on overall rehabilitation progress. Additionally, exercise adherence and motivation could be increased by integrating a conversational agent (i.e., chatbot) that educates users on relevant topics (e.g., healthy lifestyle) and provides personalized motivational messages, as well as real-time exercise support, monitoring, and feedback as previously shown for physiotherapy patients and home exercise in a hands-free augmented reality environment (Kowatsch et al., 2021). Building a strong “virtual” working alliance between a chatbot interface and a patient might prove a promising tool to improve the acceptance of the connected RehabGym and avoid mental/social distress caused by isolation. However, it should certainly not replace regular in-person controls with healthcare practitioners if possible.

In controlled clinical settings (non-connected) RehabGym concepts with complementary upper limb robotic devices have proved feasible for stroke patients with moderate to severe upper limb impairment (Lo et al., 2010; Hesse et al., 2014; Bustamante Valles et al., 2016). These studies, however, did not directly investigate how to personalize therapy plans to best leverage on a set of complementary devices, nor did they investigate the feasibility of transferring such technologies to the home of patients. Building on our previous studies, we envision that moving to such a connected RehabGym to home settings and additionally targeting more distal upper limb components should be more suited for patients with mild to moderate upper limb impairment, and without severe cognitive deficit (Lambercy et al., 2011; Chua et al., 2018; Ranzani et al., 2020).

### Potential Implementation Barriers

While promising, digital technologies will not solve all problems in the delivery of neurorehabilitation service, nor will they completely replace face-to-face visits. The idea of a connected RehabGym is to provide a new complementary model to existing rehabilitation approaches. The provision of minimally supervized therapy to neurological patients will raise a new set of questions that will need to be carefully addressed for successful implementation.

The selection of suitable patients and pairing with adequate technology-assisted therapies are necessary to ensure positive delivery and experiences of such a minimally supervized therapeutic model. Clinical considerations include patient impairment severity, medical fitness and motivation, desired rehabilitation goals, and available social supports. Certain groups of patients might find it difficult to accept this new paradigm of care, in particular the cognitively impaired, visually challenged, elderly and technologically non-savvy.

Ethical concerns may arise from patients and their families at several levels when a connected technology is introduced in the home environment, for example with respect to safety, access, privacy, data protection and respect for autonomy (Cavoukian et al., 2010). There would be anticipated needs to provide heightened cybersecurity infrastructure between healthcare institutions and technology providers, as patients’ anonymized data may need to be accessed by remote servers for processing and continuous refinement of cAI algorithms. In general, ethical frameworks related to the use of cAI for decision making in the context of healthcare are still in their infancy, and should be carefully studied in view of the increasing amount of connected tools generating health-related data (Magrabi et al., 2019).

Finally, the adoption of technology in and out of clinic may not always hinge on hard evidence or clinical effectiveness, but rather technology robustness, subjective preferences, or technological proponents and partnerships (Backus et al., 2010; Turchetti et al., 2014; Chua and Kuah, 2017). Access to rehabilitation technology in low- and middle-income countries also needs to be considered and the proposed connected RehabGym would need to be adapted for low-resource environments with less robust infrastructure (e.g., power, internet) and limited access to rehabilitation services. Cost considerations, and in particular billing and reimbursement models for such novel means of neurorehabilitation delivery should also be carefully studied to ensure its viability on a large scale. In particular, possible device rental models should be explored to minimize the treatment costs for the individual and further promote flexibility in therapy plan adjustment.

## Conclusion

Technology plays a key role in times of the COVID-19 pandemic for solving problems in essential healthcare delivery such as in neurorehabilitation. We proposed an approach to implement neurorehabilitation from a distance, through the use of digital connected interventions (e.g., minimally supervized robot-assisted therapy) that could accompany stroke patients along the continuum of care, from the hospital to their home.

For technology-based models of neurorehabilitation from a distance to become successful, three factors are crucial for their implementation: firstly, the technologies need to meet technical requirements such as robustness, safety and usability since patients train with at least one device at home (i.e., the most suitable device according to patients’ needs). Secondly, rehabilitation technologies should be scalable (i.e., easily applicable to the increasing number of patients in need of such treatment, which implies social, technical, economical and infrastructure considerations) in order to be impactful. Thirdly, the implementation of artificial intelligence embedded in neurorehabilitation technologies needs to be clinically motivated and transparent to patients, caregivers and healthcare practitioners in order to increase the trust in technology-assisted rehabilitation in a home-centered model. All these aspects are essential to ensure that neurological patients accept rehabilitation technologies and actively self-engage in therapy (Neibling et al., 2021).

The proposed model of neurorehabilitation is likely to impact neurorehabilitation beyond the COVID-19 pandemic, by providing broad access to sustained, high-quality and high-dose therapy to maximize long-term functional outcomes and promote stroke survivors’ independence and quality of life. Such a paradigm shift is bound to happen, and COVID-19 may act as an accelerator for the adoption by patients, caregivers and rehabilitation practitioners and for the market penetration of the proposed technology-assisted rehabilitation (Keesara et al., 2020). However, such a new approach to stroke rehabilitation can only become successful in the future if it is accompanied by a holistic digital transformation of healthcare systems, including appropriate responses by authorities, healthcare providers, and insurance companies.

## Data Availability

The original contributions presented in the study are included in the article/Supplementary Material, further inquiries can be directed to the corresponding authors.
